# Prognostic analysis in children with focal cortical dysplasia II undergoing epilepsy surgery: Clinical and radiological factors

**DOI:** 10.3389/fneur.2023.1123429

**Published:** 2023-03-06

**Authors:** Siqi Zhang, Yi Luo, Yilin Zhao, Fengjun Zhu, Xianping Jiang, Xiaoyu Wang, Tong Mo, Hongwu Zeng

**Affiliations:** ^1^Shantou University Medical College, Shantou University, Shantou, China; ^2^Department of Radiology, Shenzhen Children's Hospital, Shenzhen, China; ^3^Department of Epilepsy Surgical Department, Shenzhen Children's Hospital, Shenzhen, China; ^4^Department of Pathology, Shenzhen Children's Hospital, Shenzhen, China

**Keywords:** focal cortical dysplasia, radiological findings, pediatrics, prognosis, predictors

## Abstract

**Objective:**

The aim of this study was to investigate the value of clinical profiles and radiological findings in assessing postsurgical outcomes in children with focal cortical dysplasia (FCD) II while exploring prognostic predictors of this disease.

**Methods:**

We retrospectively reviewed 50 patients with postoperative pathologically confirmed FCD II from January 2016 to June 2021. The clinical profiles and preoperative radiological findings were measured and analyzed. The patients were classified into four classes based on the Engel Class Outcome System at the last follow-up. For the analysis, the patients were divided into two categories based on Engel I and Engel II–IV, namely, seizure-free and non-seizure-free groups. Qualitative and quantitative factors were subsequently compared by groups using comparative statistics. Receiver operating characteristic (ROC) curves were used to identify the predictors of prognosis in children with FCD II.

**Results:**

Thirty-seven patients (74%) had Engel class I outcomes. The minimum postsurgical follow-up was 1 year. At the epilepsy onset, patients who attained seizure freedom were older and less likely to have no apparent lesions on the preoperative MRI (“MRI-negative”). The non-seizure-free group exhibited a higher gray matter signal intensity ratio (GR) on 3D T1-MPRAGE images (*p* = 0.006), with a lower GR on T2WI images (*p* = 0.003) and FLAIR images (*p* = 0.032). The ROC curve indicated that the model that combined the GR value of all MRI sequences (AUC, 0.87; 95% CI, 0.77–0.97; *p* < 0.001; 86% sensitivity, 85% specificity) was able to predict prognosis accurately.

**Conclusion:**

A lower age at the onset or the MRI-negative finding of FCD lesions suggests a poor prognosis for children with FCD II. The model consisting of GR values from three MRI sequences facilitates the prognostic assessment of FCD II patients with subtle MRI abnormalities to prevent worse outcomes.

## 1. Introduction

Focal cortical dysplasia (FCD) is defined as a localized malformation of cortical development caused by disturbances in neural cell proliferation, migration, and differentiation (1). It is the most common cause of drug-resistant epilepsy in children, accounting for more than 30% (2). In 2011, the International League Against Epilepsy (ILAE) classified FCD into three types based on histopathological features, among which type II is characterized by disrupted cortical lamination and specific cytologic abnormalities (3). Approximately 29%−39% of children with FCD who undergo epilepsy surgery are diagnosed as type II (4), so an accurate assessment of its prognosis is much needed.

Previous research on the influencing factors of surgical outcomes in patients with intractable epilepsy due to FCD has mostly focused on the analysis of clinical indicators such as seizure forms, electroencephalography, and surgical approaches. A complete resection of the assumed epileptogenic area was the only well-established factor for a satisfactory prognosis (5–7). The radiological studies related to FCD have been mainly committed to the summary of MRI features (8, 9). Few studies have analyzed MRI findings correlated with the prognosis of FCD II, and only the absence of clear MRI findings (“MRI-negative”) has been consistently associated with a less favorable outcome (10–13). Therefore, we conduct this study to explore the relationship between clinical features, preoperative MRI manifestations, and the prognosis of postoperative epilepsy for children with FCD II to provide more objective indicators for clinical decision-making on surgical options.

## 2. Materials and methods

### 2.1. Patient selection

**Figure 1:**
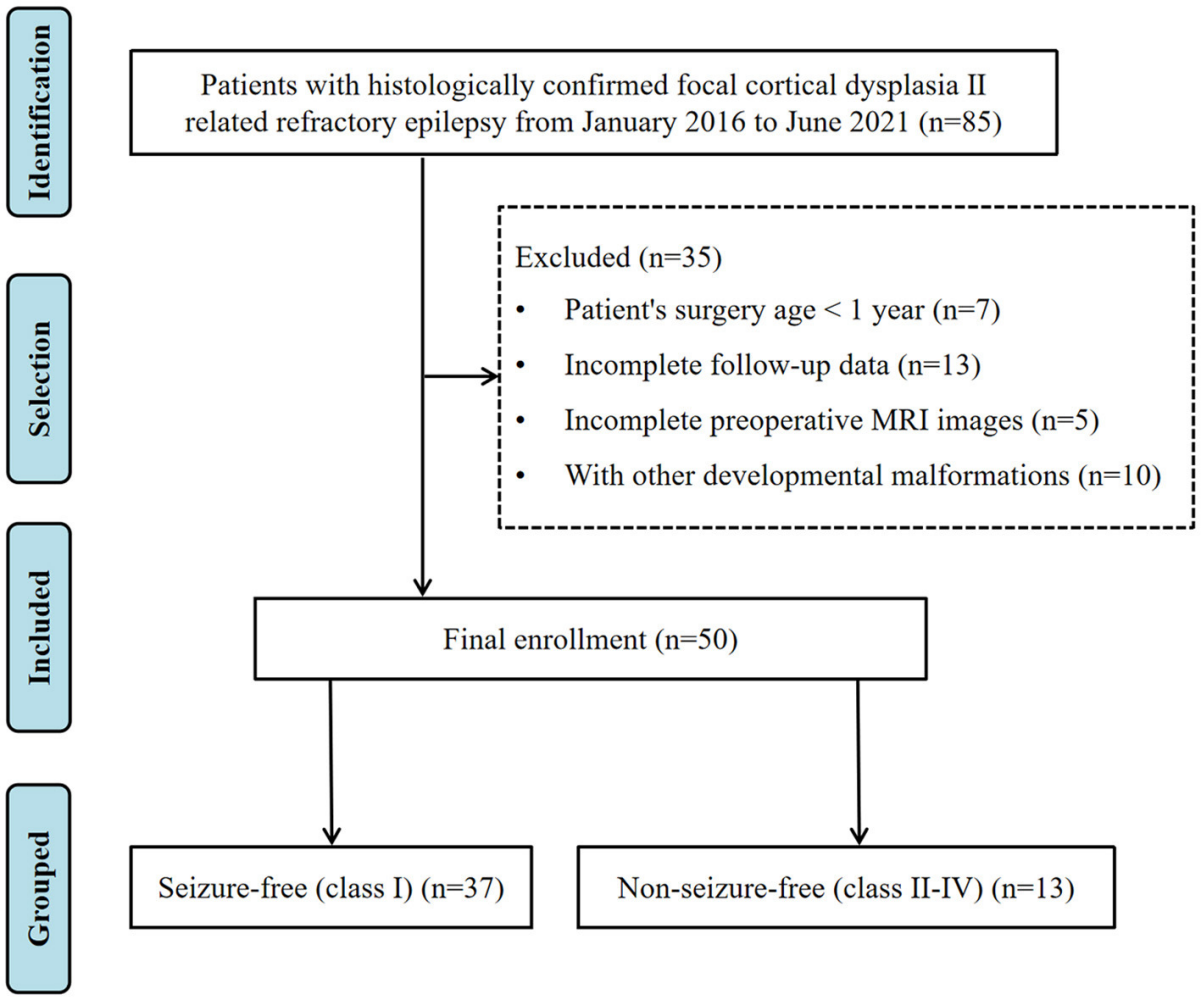
Flowchart of patient selection.

We retrospectively reviewed patients who had undergone surgical treatment for refractory epilepsy and were confirmed with a pathological diagnosis of FCD II at the Shenzhen Children's Hospital between January 2016 and June 2021. Patients were further selected based on the following criteria: (1) the age of the patient at the time of epilepsy surgery was between 1 and 18 years; (2) baseline clinical data were available and complete; (3) brain three-dimensional high-resolution MRI data were collected before surgery; (4) postsurgical MRI data were available; and (5) clinical follow-up was performed for at least 12 months. The exclusion criteria were incomplete follow-up data, unqualified preoperative MRI images, or combined with other developmental malformations (such as tuberous sclerosis, hemispherical dysplasia, and periventricular nodular heterotopia). We also excluded children who had undergone surgery before reaching 1 year of age. Although myelination in the temporal lobe does not fully mature until 2 years old, we used the age of 1 year as a cutoff because our cohort did not include patients whose lesions were in the temporal lobe. A total of 50 children were finally included in this study. This study was approved by the local institutional review board. Figure 1 shows the workflow for patient selection.

### 2.2. MRI acquisition

The MRI investigations were conducted on a Siemens 3.0T superconducting high-field magnetic resonance imaging system (Skyra, Siemens, Germany) with a dedicated epilepsy protocol, including a 3D T1-weighted gradient-echo sequence (MPRAGE; axial acquisition; TR: 2,000 ms; TE: 2.4 ms; slice thickness 1.0 mm), TSE T2WI sequence (axial acquisition; TR: 4,500 ms; TE: 106 ms; slice thickness 2.0 mm), and TSE FLAIR T2WI sequence (axial acquisition; TR: 13,000 ms; TE: 134 ms; slice thickness 2.0 mm).

### 2.3. Clinical profiles

Baseline presurgical clinical profiles, pathologic data, and the postsurgical state were collected from the medical records. The types of presurgical seizures were divided into focal seizures and secondarily generalized seizures according to the ILAE classification and definition of epilepsy syndromes with onset in childhood (14). The diagnosis of FCD II was confirmed by pathologists according to the histologic classification system proposed by the ILAE (3). The outcome of the last follow-up was assessed according to Engel's classification. For the analysis, patients were divided into two categories only, Engel I and Engel II-IV, and named as seizure-free and non-seizure-free groups. A visible or invisible lesion was not wholly resected because of the overlap of the epileptogenic focus with the eloquent area, which was referred to as the incomplete resection (5).

### 2.4. Surgery

The patients were approved for surgery after a consensus was reached regarding the multimodality data which included clinical, neuroimaging, and electrophysiological results. All the patients underwent stereotactic EEG. Neuronavigation with MRI and PET-CT data was used to confirm the FCD location. In patients with subtle findings on MRI, the presumed location of the epileptogenic zone was identified by EEG and PET-CT data. During the operation, intracranial electrodes were placed for stereotactic EEG monitoring, and the epileptogenic and functional zones were further assessed by electrical cortical stimulation, while the lesion was resected along with 0.5 cm of the circumferential cortex to avoid the residual abnormal brain tissue.

### 2.5. Imaging review

All images were reviewed by two senior neuroradiologists with at least 8 years of working experience. The reviewers were blinded to the clinical diagnosis and pathologic outcomes. When two radiologists disagreed, the third one with 20 years of experience gave the final opinion and reached a consensus. Abnormal MRI findings were identified as definite abnormal structures or signals on 3D T1-MPRAGE, T2WI, and FLAIR images. The detailed qualitative MRI indicators were as follows: cortex thickening, gray-white matter blurring, transmantle sign, and signal intensity changes of both the gray and white matter. The definition of the transmantle sign was “a markedly hyperintense funnel-shaped subcortical zone tapering toward the lateral ventricle” (15). The MRI-negative was defined as the invisible abnormal signals of the cortical or subcortical part.

RadiAnt DICOM Viewer (2021.2.2) post-processing software was used for the quantitative measurement of FCD II target lesions. The target lesion was used to indicate the epileptogenic area planned for resection. The regions of interest (ROIs) were referred to as the area where the quantitative MRI parameters were measured and the area of the sample to be sent for subsequent pathological verification. The detailed measurement programs were as follows. First, preoperative MRI found the abnormal signal region, which was confirmed by SEEG and PET-CT as an epileptogenic focus, and then it was marked as a “target lesion” on the neuronavigation images (which could correspond to the preoperative 3D MRI images). On the largest slice of the target lesion, the definite gray matter and definite white matter areas with relatively homogeneous signals were selected to draw ROIs, respectively (the measuring area of each ROI was ~10 mm^2^), the ROI areas were resected, and the lesions were confirmed by pathology. When the abnormal signal area found by preoperative MRI was extensive or included functional areas, we avoided eloquent areas with SEEG and marked the rest of the part as a “target lesion.” On the largest slice of the target lesion, the definite gray matter and definite white matter areas with relatively homogeneous signals were selected to draw ROIs, respectively, excised with accurate spatial localization, and the lesions were confirmed by pathology. When no significant abnormal lesions were found on preoperative MRI, PET-CT images and SEEG were used to determine the “target lesion,” which would also mark on the neuronavigation images. On the largest slice of the target lesion, the definite gray matter and definite white matter areas with relatively homogeneous signals were selected to draw ROIs, respectively, the ROI areas were resected, and the lesions were confirmed by pathology.

**Figure 2:**
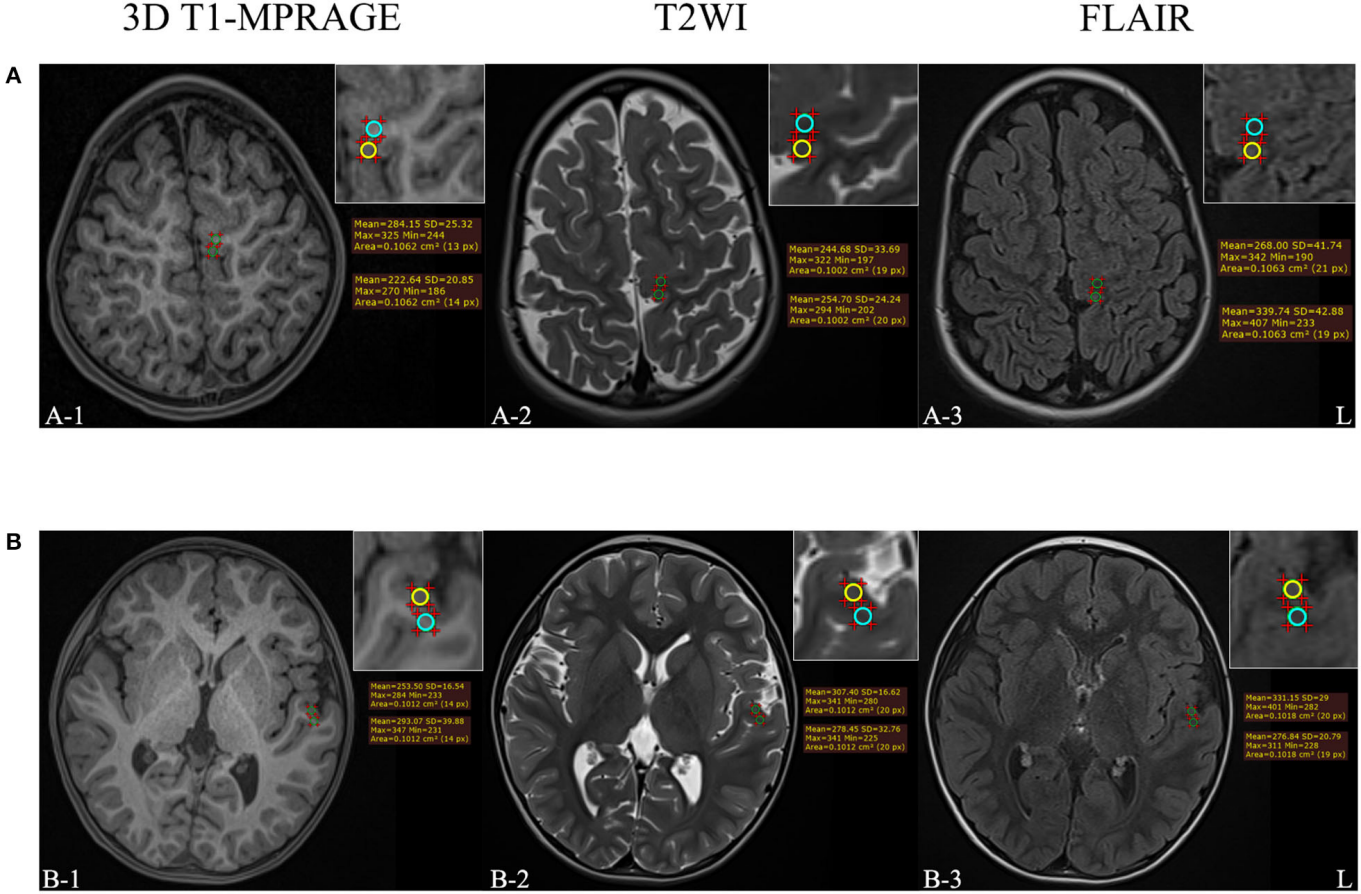
**(A)** The example of MRI-positive images of a 3-year-old female patient who was diagnosed with FCD IIb. **(B)** The example of MRI-negative images of a 4-year-old male patient who was diagnosed with FCD IIa. The upper right corner of each image was a partial enlargement. The signal intensity in the gray and white matter regions of the target lesion was measured, respectively, and the yellow circle means the ROI of abnormal gray matter while the blue circle means the ROI of abnormal white matter.

The dorsal thalamus was selected as the reference, and its mean signal intensity value was used to standardize the measured values of the target lesion, for eliminating individual differences. The gray matter signal intensity ratio (GR) and the white matter signal intensity ratio (WR) of FCD II lesions on 3D T1-MPRAGE, T2WI, and FLAIR images were calculated. The MRI features and parameter measurement process of patients with FCD II are shown in Figure 2.

### 2.6. Statistical analysis

SPSS 18.0 (IBM, New York, USA) statistical analysis software was used for data analysis. Continuous variables are described as mean (standard deviation, SD) or median (interquartile range, IQR), and categorical variables are presented as frequencies (%). The independent sample *t*-test or the Mann–Whitney *U*-test was used to analyze continuous variables, and the chi-square or Fisher's exact test was used for categorical variables. A *p*-value of <0.05 was considered to be statistically significant. Receiver operating characteristic (ROC) curves were used to analyze and determine the best MRI parameters for predicting the prognostics of children with FCD II. Diagnostic efficacy including sensitivity and specificity was calculated at the optimal cutoff value.

## 3. Results

### 3.1. Clinical characteristics and radiological features

Table 1 shows a comparison of the clinical characteristics and radiological features between the seizure-free and non-seizure-free groups. A total of 50 patients were included in the present study. The subjects consisted of 26 male patients and 24 female patients, and the mean age of the patients at the time of epilepsy onset was 2.08 years (SD, 2.49). The duration of epileptic symptoms was 4.13 years (SD, 4.23). Of the patients, 38 had focal seizures, 33 had lesions located in the frontal lobe, and 38 obtained the completed resection. According to the ILAE, 22 cases were histopathologically diagnosed with FCD IIa, and the rest were diagnosed with FCD IIb. The median follow-up time was 22 months (IQR, 15–33). In the entire postoperative period, 37 patients (74%) belonged to Engel class I. Patients who attained seizure freedom were older at the time of epilepsy onset (mean age of 2.4 years vs. 1.2, *p* = 0.042).

**Table 1:** Comparison of clinical characteristics and radiological features between the seizure-free and non-seizure-free groups.

**Variables**	**Seizure-free (*n* = 37)**	**Non-seizure-free (*n* = 13)**	***p*-value**
Age at epilepsy onset (years), mean (SD)	2.4 (2.8)	1.2 (1.2)	0.042
Duration of epilepsy (years), mean (SD)	3.9 (3.7)	4.8 (5.5)	0.502
Male sex, *n* (%)	19 (51)	7 (54)	0.877
**Seizure types**, ***n*** **(%)**			0.624
Focal seizures	28 (76)	10 (77)	
Secondarily generalized seizures	9 (24)	3 (23)	
**Lesion location**, ***n*** **(%)**			0.277
Frontal lobe	13 (35)	2 (15)	
Non-frontal lobe	3 (8)	2 (15)	
Multiple lesion	21 (57)	9 (70)	
Complete resection, *n* (%)	31 (84)	7 (54)	0.040
**Histology**, ***n*** **(%)**			0.139
FCD IIa	14 (38)	8 (62)	
FCD IIb	23 (62)	5 (38)	
**Radiological features**, ***n*** **(%)**			
Cortex thickening	30 (81)	8 (62)	0.149
Gray-white matter blurring	22 (59)	5 (38)	0.191
The transmantle sign	10 (27)	2 (15)	0.331
Abnormal signal of gray matter	16 (43)	3 (23)	0.170
Abnormal signal of white matter	15 (41)	6 (46)	0.724
MRI-negative	2 (5)	4 (31)	0.033

Cortex thickening was detected in 38 patients (76.0%), followed by gray–white matter blurring which was detected in 27 patients (54.0%). The transmantle sign was merely found in 12 (24.0%) patients with FCD IIb. Compared to the seizure-free children, neither the gray matter nor the white matter signal changes of the lesions were apparent on the MRI images of the non-seizure-free children. MRI was considered negative in two patients (5%) in the group with seizure-free outcomes vs. four patients (31%) in those with non-seizure-free outcomes (*p* = 0.033).

### 3.2. MRI parameters and prognosis prediction

The MRI 3D T1-MPRAGE GR of FCD II lesions with the seizure-free group was lower than that with the non-seizure-free group, and the difference was statistically significant (mean value of 0.65 vs. 0.74, *p* = 0.006). The MRI T2WI GR (mean value of 1.37 vs. 1.19, *p* = 0.003) and FLAIR GR (mean value of 1.27 vs. 1.08, *p* = 0.032) of FCD II lesions in the seizure-free group were higher than that in the non-seizure-free group. The difference in WR values on three MRI series images was not significant between the two groups.

**Figure 3:**
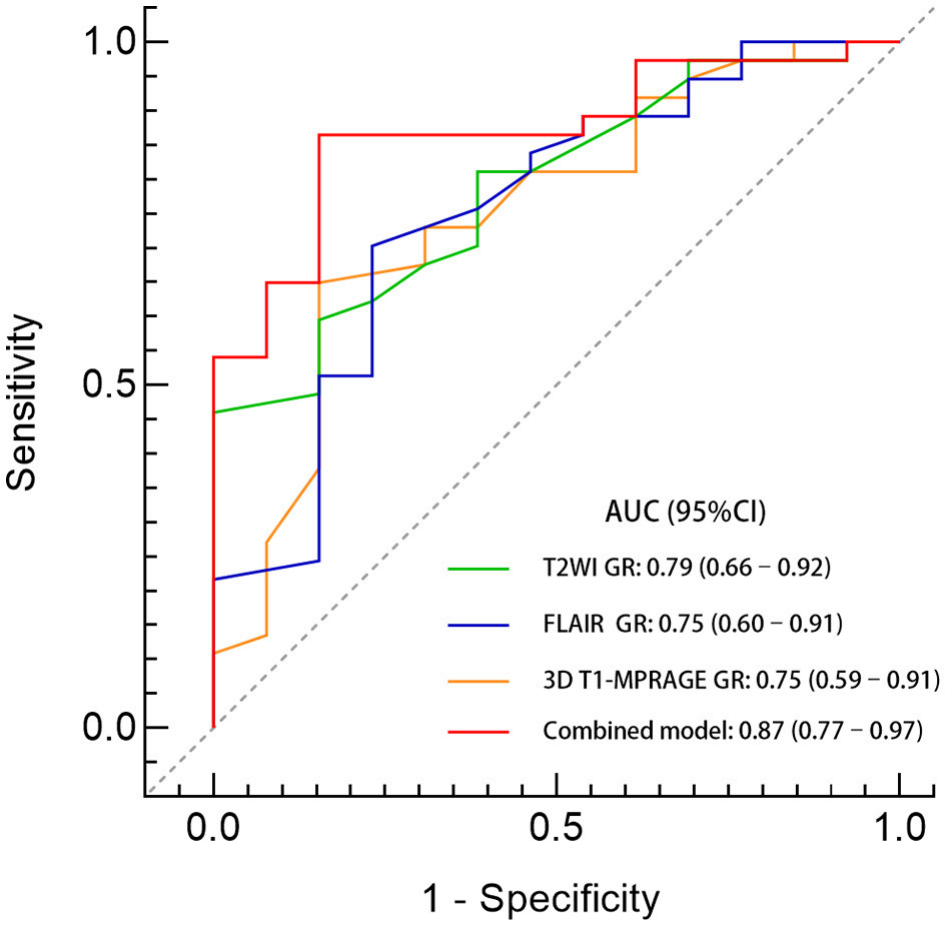
Comparison of predictive performance between the GR values of different sequences. A GR ratio of <0.69 on 3D T1-MPRAGE images combined with a GR ratio of > 1.34 on T2WI images and a GR ratio of >1.12 on FLAIR images identified those with a seizure freedom outcome. The AUC of the combined model was 0.87 (95% CI, 0.77–0.97; *p* < 0.001) with a sensitivity of 86% and a specificity of 85%.

The ROC curves were used to analyze the predictive value of MRI parameters for postoperative outcomes. The area under the curve (AUC) for GR on 3D T1-MPRAGE, T2WI, and FLAIR images were 0.75 (95% CI, 0.59–0.91), 0.79 (95% CI, 0.66–0.92), and 0.75 (95% CI, 0.60–0.91), respectively. At a cutoff GR value of 0.69, the sensitivity and specificity of the 3D T1-MPRAGE images were 65% and 85%. At a cutoff GR value of 1.34, the sensitivity and specificity of the T2WI images were 60% and 85%. At a cutoff GR value of 1.12, the sensitivity and specificity of the FLAIR images were 70% and 77%. The model combining all three GR values had the highest AUC (0.87; 95% CI, 0.77–0.97; *p* < 0.001; 86% sensitivity, 85% specificity). The details are shown in Table 2 and Figure 3.

**Table 2:** Comparison of MRI parameters between the seizure-free and non-seizure-free groups.

**MRI parameters**	**Seizure-free (*n* = 37)**	**Non-seizure-free (*n* = 13)**	***t***	***p*-value**
**3D T1-MPRAGE, mean (SD)**				
GR	0.65 (0.10)	0.74 (0.10)	−2.873	0.006
WR	0.86 (0.21)	0.93 (0.17)	−1.088	0.282
**T2WI, mean (SD)**				
GR	1.37 (0.20)	1.19 (0.11)	3.121	0.003
WR	1.20 (0.40)	1.00 (0.25)	1.643	0.107
**FLAIR, mean (SD)**				
GR	1.27 (0.30)	1.08 (0.13)	2.206	0.032
WR	1.07 (0.36)	0.95 (0.27)	1.018	0.314

GR, gray matter signal intensity ratio; WR, white matter signal intensity ratio.

## 4. Discussion

FCD is a subtype of cortical dysplasia and the most common cause of medically refractory epilepsy in children (2). Persistent seizures in childhood, as well as the long-term usage of multiple antiepileptic drugs, have significant negative effects on children's growth, development, and cognitive functions. Previous studies have shown (5–7) that early surgery and the complete resection of the epileptogenic zone draw support from the plasticity of brain development to provide more opportunities for nerve cells to compensate, which is more beneficial for postoperative recovery and to improve the quality of life in children (16). We obtained consistent results from the analysis of clinical data that complete resection was beneficial for patient prognosis. MRI is currently the best method for non-invasive preoperative assessment of FCD, which helps to clarify the relationship between the lesion site, boundary, and surrounding tissues (8), and provides a basis for satisfiable surgical decision-making. Therefore, the screening of key clinical and MRI findings related to prognostic grading is of great significance for preoperative personalized diagnosis, treatment evaluation, and postoperative life quality improvement in children with FCD II.

The statistical results of clinical data showed that the prognosis of children with earlier seizures was poor. The results of Maynard et al. (17) confirmed that age was an independent risk factor for seizure severity, with drug-resistant epilepsy occurring earlier (*p* < 0.001). Another cohort study of 112 young children (<6 years old) with FCD II (18) showed that young children are more prone to diffuse abnormal EEG discharges and spastic seizures due to immaturity of the brain, resulting in moderate or even severe psychosis developmental delay. This suggests that clinical attention should be paid to the early screening of children with FCD II, especially the younger ones, and the early complete surgical resection of the malformed cortex could achieve a better prognosis.

In this cohort of patients, ~12.0% (6/50) had negative preoperative MRI results, which is consistent with the data reported in previous studies that ~15%–30% of patients with type II showed no definite abnormality in MRI (3, 19). Among them, the poor prognosis group accounted for 30.0% (4/13), and the postoperative pathological results were all type IIa. The MRI features of FCD IIa are not typical, often showing mild structural changes such as focal cortical thickening and unclear gray–white matter demarcation, and the probability of MRI-negative is much higher than that of type b (20). In addition, type IIa is more likely to lead to widespread epileptiform abnormal discharges (21), which makes it difficult to determine the boundary of the lesion with intraoperative EEG monitoring. All these factors may lead to the postoperative recurrence of epilepsy in children. Therefore, we believe that incomplete resection of potential epileptogenic focus caused by negative MRI is an important factor in the prognostic evaluation of children with FCD II, which is consistent with most previous studies (10–13).

MRI-negative is a misnomer that comprises patients without an MRI lesion or with a subtle MRI lesion but being overlooked (8). Although high-field imaging and postprocessing techniques have dramatically improved the detection rate of FCD, there are still limitations in the artifacts (22), the high false positive rate outcome (23), and clinical application promotion. Due to the preferential occurrence of dysmorphic neurons in deep cortical layers (24, 25), MRI abnormalities are mostly displayed close to the gray matter–white matter interface. Partial volume effects interfere with the identification of signal changes, but quantitative analysis help to reflect different features of the signal objectively. Considering the matter of availability and ease of use, this study calculated ratios based on direct measurement and found that GR holds significant potential for predicting prognosis. The results of Donkels et al. (26) indicated that the abnormal proliferation and differentiation of oligodendrocyte precursor cells caused barriers to form gray matter myelination in FCD IIa, which might contribute to the epileptogenicity of this cortical malformation. This may explain why the GR was more sensitive to prognosis than the WR.

Furthermore, compared to the other two sequences, the GR on T2WI images had the highest AUC. House et al. (27) reported similar results: concerning the visualization of FCD by highlighting blurring of the gray matter–white matter junction, the morphometric analysis of T2WI MRI data on average was superior to T1WI-based morphometry (*p* < 0.003). Even in clinical MRI-negative cases, the quantitative approach on T2WI can help to distinguish the lesions and perilesional areas (28). Notably, the advantage of the combined model was more prominent (AUC, 0.87; 95% CI, 0.77–0.97, *p* < 0.001). Moreover, the objectivity of quantitative indicators could also help reduce the anxiety of parents, which may facilitate the implementation of surgeons' surgical proposals. Therefore, we suggest the application of multimodal MRI profiling and prospectively validate these MRI-based ratios in subsequent children undergoing epilepsy surgery for FCD II to improve the prognosis of these patients.

This study has several potential limitations. First, it has limitations inherent to the retrospective study design and manual measurement. Second, there are some variations in the number of cases with different prognoses for surgery and in the data for positive or negative MRI findings, which limit the interpretation of the results. Third, since the principle of avoiding functional area resection was hard to violate, the cohort existed for incomplete resection cases. Finally, due to the enrollment time limitation, some children merely meet the basic follow-up requirements of more than 1 year, while the possibility of epilepsy recurrence cannot be ruled out. These issues need to be further explored in future.

## 5. Conclusion

This study aimed to enhance the understanding of radiological findings in the prognostic analysis for children with FCD II. Based on the statistical results, we demonstrated that lesser onset age and the MRI-negative finding of FCD lesions could be poor prognostic predictors. The model consisting of GR values from three MRI sequences may facilitate the prognostic assessment of those patients with subtle radiological features to prevent worse outcomes. Further studies are needed to validate the predictive value of this model for other types of cortical malformation.

## Data availability statement

The original contributions presented in the study are included in the article/Supplementary material, further inquiries can be directed to the corresponding author.

## Ethics statement

The studies involving human participants were reviewed and approved by the Medical Ethics Committee of the Shenzhen Children's Hospital (2022-08-602). Written informed consent to participate in this study was provided by the participants' legal guardian/next of kin.

## Author contributions

SZ collected, analyzed the data, and wrote the whole manuscript. YL reviewed the methods. FZ and XJ improved the clinical data. XW and TM reviewed and measured all MRI data. YZ finished the project administration. HZ revised and made final approval of this manuscript. All authors contributed to the article and approved the submitted version.
