# Supplementary material for: Prognostic analysis in children with focal cortical dysplasia II undergoing epilepsy surgery: Clinical and radiological factors
Source: Front Neurol. 2023 Mar 6;14:1123429. doi: 10.3389/fneur.2023.1123429 (PMC10025379; doi:10.3389/fneur.2023.1123429)
Supplement: Supplementary file 1 [file Table_1.docx]

Supplementary Material

Prognostic analysis in children with focal cortical dysplasia II undergoing epilepsy surgery: Clinical and radiological factors

Siqi Zhang, Yi Luo, Yilin Zhao, Fengjun Zhu, Xianping Jiang, Xiaoyu Wang, Tong Mo and Hongwu Zeng^*^

*** Correspondence:** Corresponding Author: [homerzeng@126.com](mailto:homerzeng@126.com)

# Supplementary Tables

**Supplementary Table 1.** Comparison of SEEG between the seizure-free and non-seizure-free.

| **Variables** | **Seizure-free**  **(n = 37)** | **Non-seizure-free**  **(n = 13)** | ***p* -Value** |
| --- | --- | --- | --- |
| SEEG, n (%) |  |  | 0.086 |
| MRI-negative | 2 (5) | 4 (31) |  |
| MRI-positive, discordant non-invasive investigation | 23 (62) | 6 (46) |  |
| MRI-positive, define extent of the lesion | 12 (33) | 3 (23) |  |
